# Worse sleep quality predicts early drop out from physical exercise programs

**DOI:** 10.5935/1984-0063.20220078

**Published:** 2022

**Authors:** Flávio Augustino Back, Adriano Akira Ferreira Hino, Wilynson Gomes Bojarski, Eduardo Henrique Rosa Santos, Leandro dos Santos Afonso, Lucélia Justino Borges, Fernando Mazzilli Louzada

**Affiliations:** 1 Federal University of Paraná, Physiology, Human Chronobiology Lab - Curitiba - Paraná - Brazil; 2 Pontifícia Universidade Católica do Paraná, Health Sciences Graduate Program, School of Medicine - Curitiba - Paraná - Brazil; 3 Federal University of Uberlândia, Faculty of Physical Education and Physiotherapy - Uberlândia - Minas Gerais - Brazil; 4 Education and Research Institute Alfredo Torres, - Campo Grande - Mato Grosso do Sul - Brazil; 5 Federal University of Paraná, Department of Physical Education - Curitiba - Paraná - Brazil

**Keywords:** Sleep, Exercise, Behavior, Adult, Fitness Centers

## Abstract

**Introduction and Objective:**

Sleep quality (SQ) benefits from regular physical exercise (PE) practice, but the effect of SQ over behavioral aspects of PE is not well known. In this study, we tested whether sleep variables can predict the drop out risk for PE programs during a six-week critical period for habit formation at gyms.

**Material and Methods:**

We assessed 153 volunteers, freshly enrolled at three different gyms and from both sexes, with average age of 33.6 (±11.9) years. Questionnaires provided sociodemographic, health, sleep, physical activity and circadian rhythmicity information. Daily PE practice frequency was monitored using the gym’s turnstiles electronic records. We created a multivariate model using Cox regression in order to test the risk of PE program drop out during the first six weeks.

**Results:**

Worse SQ predicted a higher drop out risk (HR=1.11; 95%CI = 1.02-1.21; *p*<0.05), even when adjusted for other potential confounding variables.

**Conclusion:**

We found that worse SQ predicted a higher early drop out from PE programs in the formal context of gyms during the first six weeks, along with other variables related to PE practice.

## INTRODUCTION

Sleep and physical activity (PA) are modifiable behaviors that influence each other and can predict health outcomes - in which poor sleep/PA habits are linked with increased mortality risk^1,2^. Physical exercise (PE) - the regular and structured practice of PA with the aim to keep or improve one’s physical condition - is known as a non-pharmacological intervention capable of improving sleep parameters with a broad literature on this subject^3,4^.

Typical PE (≥4 weeks) shows a consistent improvement in the subjective sleep quality (SQ)^5^. In contrast, poor sleep quality predicted reduced daily or total physical activity^6^. However, we do not know of any published results about the possible predictive role of sleep in behavioral aspects of PE.

For this reason, our aim was to test whether sleep variables could predict the drop out risk for PE programs at gyms during a six-week evaluation period. This period seems critical to habit formation of PE at gyms^7^.Our hypothesis was that worse sleep quality predicts a higher risk of dropping out of PE programs.

## MATERIAL AND METHODS

### Study design and sample

We studied 153 new clients from three different gyms in Curitiba (PR/Brazil) using a longitudinal observational design. They were tracked for at least 3 months, between February and September 2019. For this study, we used only the six-week follow-up period.

The gyms were selected based on convenience, with 500 to 1,400 registered clients each. The monthly fee guaranteed access to all offered activities, which were supervised by physical education professionals and included resistance exercises, aerobics, and group training modalities. The gyms were open in the morning and evening (6 a.m. to 11 p.m.) during weekdays and in the morning and afternoon (8 a.m. to 4 p.m.) during weekends. The gyms did not provide any other services besides the practice of physical exercise.

The sample consisted of freshly enrolled gym clients. Inclusion criteria were: attending the gym for less than two weeks; age between 18-65 years old. Exclusion criteria were: being pregnant or lactating; being a night worker or shift worker; being under personalized care (personal trainer). A total of 212 individuals participated in the research, from which we excluded 51 for not completing the questionnaires; 5 for presence data not registered by the turnstile or inconsistency of the first presence; 2 for not living in the city; and 1 for being under treatment for chemical dependency.

The study was approved by the ethics committee for research in human beings of the Federal University of Paraná (opinion 80184517.2.0000.0102, approved in 2018) and all participants were informed and signed a consent form.

### Data collection procedure

The recruited subjects’ evaluations were carried out by researchers and took place up to two weeks after the registration of the first attendance at the gym. Demographic, health, PA, sleep, and chronobiological information were collected through questionnaires sent through a messaging application (WhatsApp). Body mass, height, and abdominal perimeter measurements were performed using an portable digital scale (Wizo^®^), an inelastic measuring tape on a vertical surface to 100 cm point distant of the ground and an inelastic, millimeter, anthropometric tape measure, respectively.

Volunteer’s practice frequency was monitored with the help of an electronic turnstile system for over 3 months following their gym enrollment. When practice was either interrupted for four weeks or the contracted plan was canceled, the participant was classified as a dropout. Those who had interruptions shorter than four weeks were classified as non-dropouts.

### Tools and data treatment

#### Circadian rhythmicity and sleep aspects

SQ was measured by the Pittsburgh sleep quality index (PSQI)^8^, validated for the Brazilian population^9^, which assesses the SQ of the last 30 days with 19 questions categorized into seven components with scores ranging from zero to three. The sum of the components generates a score that ranged between 0 and 21; higher scores indicate worse SQ.

The morningness-eveningness questionnaire (MEQ)^10^ validated for the Portuguese language^11^, was used to assess daytime preference. We analyzed the MEQ as a continuous variable (scores ranging from 16 to 86; higher scores indicating greater morningness, and smaller scores greater eveningness).

Data corresponding to sleep time on working and free days, social jetlag, sleep debt and mid-point of sleep corrected for weekly sleep deficit was assessed using the Munich chronotype questionnaire (MCTQ)^12^. The Portuguese language version was made available by the authors in 2017.

#### Physical activity and exercise frequency

We used the short version of the international physical activity questionnaire (IPAQ), validated for the Brazilian population^13^, to measure the total PA level, in order to control the global PA performed outside the gym. By calculating the weekly energy expenditure in metabolic equivalents, we created a binary variable based on the recommendations (<600 mets-minutes and ≥600 mets-minutes per week).

The registration of attendance was taken as a sign of the PE practice being executed, since no other activities were available at the site. The activities were prescribed and supervised by physical education professionals from the gyms. We did not oversee the type, duration, or intensity of exercises performed by participants.

#### Data analysis

Central tendency measures and dispersion were used to describe the quantitative variables while frequency distribution (absolute and relative) was used with qualitative variables. In order to assess the drop out risk during the first six weeks we performed a Cox proportional hazards model considering demographic, health, PA, sleep, chronobiological, and gym-related characteristics. A multivariate model was developed using Cox regression, integrating the explanatory variables that presented a significance level <0.05. We performed all analyses using the IBM SPSS Statistics version 20.0 program, assuming a statistical significance of *p*<0.05.

## RESULTS

Table 1 describes the general characteristics of the sample, according to the PE programs dropout from up to the sixth week. The sample consisted of adults with average age of 33.6 (±11.9) years and average body mass index of 26.0 (±4.5) kg/m^2^, with no difference between men and women. Most of the sample was composed of women (66.7%), working (83.7%), single (53.6%), had no children under 18 (68%), had at least completed university (55.6%), working and/or studying at least 36 hours a week (66.2%), had good self-perception of health (55.6%), had no chronic diseases (85.7%), non-smoker (93.5%) and used to drink alcoholic beverages (64.1%).

**Table 1:** Sample characteristics according to sociodemographic, health, behavioral, physical activity, sleep, circadian rhythmicity variables, categorized into non-dropouts and dropouts in the first six weeks of the gym, and association with the outcome of dropout. Curitiba - Brazil, 2019.

Variables	Total		Non-dropouts		Dropouts		Crude Analysis	
	n	%	n	%	n	%	HR	95%CI
**Total**	153	100	111	72.5	42	27.5	-	-
**Demographic characteristics**								
**Sex**								
Male	51	33.3	36	70.6	15	29.4	1.14	(0.61-2.14)
Female	102	66.7	75	73.5	27	26.5	1.00	
Average age in years (± SD)	33.6	11.9	35.6 ± 12		28.4 ± 9.7		0.95^**^	(0.92-0.98)
**Educational level**								
Up to high school graduate	68	44.4	40	58.8	28	41.2	2.67^*^	(1.17-6.12)
Bachelor’s graduate	44	28.8	37	84.1	7	15.9	0.91	(0.32-2.6)
Completed post graduation	41	26.8	34	82.9	7	17.1	1.00	
**Civil status**								
Single	82	53.6	52	63.4	30	36.6	2.5^**^	(1.27-4.83)
Married/Stable union	71	46.4	59	83.1	12	16.9	1.00	
**Number of childs <18yo**								
0	104	68	73	70.2	31	29.8	2.51	(0.6-10.47)
1	33	21.6	24	72.7	9	27.3	2.26	(0.49-10.48)
2 or more	16	10.5	14	87.5	2	12.5	1.00	
**Study hours or workhours per week**								
Does not work or study	13	8.8	9	69.2	4	30.8	0.87	(0.27-2.84)
3-20h	15	10.1	12	80.0	3	20.0	0.58	(0.16-2.14)
21-35h	22	14.9	16	72.7	6	27.3	0.79	(0.28-2.22)
36-44h	35	23.6	26	74.3	9	25.7	0.73	(0.29-1.85)
45-52h	37	25	26	70.3	11	29.7	0.83	(0.34-2)
>52h	26	17.6	17	65.4	9	34.6	1.00	
**Health characteristics or associated with health** **self-perception**								
							
Bad	8	5.2	4	50	4	50	1.69	(0.49-5.76)
Regular	36	23.5	27	75	9	25	0.78	(0.29-2.1)
Good	85	55.6	63	74.1	22	25.9	0.83	(0.35-1.94)
Excellent	24	15.7	17	70.8	7	29.2	1.00	
**Presence of chronic disease**								
None	126	85.7	92	73.0	34	27.0	0.73	(0.32-1.65)
1 or more	21	14.3	14	66.7	7	33.3	1.00	
**Tobacco usage**								
No	143	93.5	103	72.0	40	28.0	1.59	(0.38-6.59)
Yes	10	6.5	8	80	2	20	1.00	
**Alcoholic beverage usage**								
No	55	35.9	39	70.9	16	29.1	1.12	(0.6-2.1)
Yes	98	64.1	72	73.5	26	26.5	1.00	
**PA characteristics or associated with gym**								
PA total								
<600 met-min per week	32	20.9	27	84.4	5	15.6	0.48	(0.19-1.22)
>=600 met-min per week	121	79.1	84	69.4	37	30.6	1.00	
**Duration of membership plan**								
1-month	47	30.9	25	53.2	22	46.8	3.66^**^	(1.68-7.95)
3-month to 6-month	44	28.9	34	77.3	10	22.7	1.57	(0.64-3.86)
Annual	61	40.1	52	85.2	9	14.8	1.00	
Frequency during week one (Average ± SD)	3.1 ± 1.4		3.3 ± 1.4		2.6 ± 1.2		0.73^**^	(0.58-0.92)
**Variables**	**Total**		**Non-dropouts**		**Dropouts**		**Crude Analysis**	
	**n**	**%**	**n**	**%**	**n**	**%**	**HR**	**95%CI**
**Gym enrollment time**								
February and March	54	35.3	36	66.7	18	33.3	1.66	(0.66-4.19)
April and May	34	22.2	23	67.6	11	32.4	1.57	(0.58-4.23)
June and July	39	25.5	32	82.1	7	17.9	0.81	(0.27-2.4)
August and September	26	17	20	76.9	6	23.1	1.00	
**Anthropometric characteristics**								
BMI, (kg/m^2^) (Average ± SD)	26 ± 4.5		25.9 ± 4.4		26.5 ± 5		1.02	(0.96-1.09)
Abdominal perimeter (cm) (Average ± SD)	89.9 ± 11.3		89.7 ± 10.9		90.4 ± 12.4		1	(0.98-1.03)
**Circadian rhythmicity and sleep characteristics**								
Diurnal preference HO (MEQ score) (Average ± SD)	53.7 ± 11.0		54.9 ± 10.6		50.7 ± 11.7		0.97^*^	(0.94-0.99)
Wake up during workdays (h)(Average ± SD)	6:54 ± 1.1		6:54 ± 1		6:54 ± 1.3		1,09	(0,82-1,45)
Bedtime during workdays (h) (Average ± SD)	23:48 ± 1.1		23:42 ± 1.1		23:54 ± 1.3		1.13	(0.88-1.46)
Wake up during free days (h)(Average ± SD)	8:54 ± 1.5		8:48 ± 1.5		9:12 ± 1.4		1.21	(0.99-1.48)
Bedtime during free days (h)(Average ± SD)	00:36 ± 1.4		00:36 ± 1.4		00:48 ± 1.5		1.13	(0.91-1.38)
MSFsc (Average ± SD)	4.4 ± 1.3		4.3 ± 1.3		4.6 ± 1.3		1.17	(0.93-1.49)
SocialJetlag (h) (Average ± SD)	1.5 ± 1.1		1.4 ± 1		1.6 ± 1.2		1.2	(0.91-1.6)
Sleep debt (h) (Average ± SD)	1.1 ± 1.3		1 ± 1.2		1.3 ± 1.6		1.15	(0.91-1.45)
Sleep duration during workdays (h)(Average ± SD)	7.1 ± 1.1		7.2 ± 1.1		7.1 ± 1.2		0.94	(0.71-1.25)
Sleep duration during free days (h)	8.3 ± 1.3		8.2 ± 1.3		8.4 ± 1.4		1.12	(0.88-1.43)
(Average ± SD)								
Sleep Quality (score) (Average ± SD)	6 ± 3.3		5.5 ± 3		7.0 ± 3.9		1.11^**^	(1.03-1.21)

Notes: HR = Hazard Ratio; CI = Confidence interval; SD = Standard deviation; yo = Years old; MEQ = Morningness-eveningness questionnaire; h = Hours; met = Metabolic equivalent;

* *p*<0.05;

** *p*<0.01.

The mean SQ score was 6 (±3.3). According to clinical criteria, scores above 5 represent poor SQ; 47.1% of the sample achieved scores above 5 (data not described in the table).

During the first six weeks, 27.5% of the sample drop out from their physical exercise programs. The dropouts had worse SQ than the non-dropouts (*p*<0.05) (Table 1).

Table 2 shows the multivariate model for dropout prediction. The model shows that those who signed up for the gym’s monthly plan had a 3.4 higher dropout risk compared to those who contracted the annual plan (HR=3.43; 95%CI = 1.53-7.68; *p*<0.01). Higher frequency during week 1 was associated with lower risk of dropping out (HR=0.66; 95%CI = 0.5-0.87; *p*<0.01). Even after adjusting for possible confounding variables, a higher Pittsburgh Sleep Quality Index score was associated with a higher risk of dropping out (HR=1.11; 95%CI = 1.02-1.21; *p*<0.05).

**Table 2:** Multivariate model for predicting PE program drop out during the first six weeks.

Variables	Multivariate model		
	HR	95%CI	p
Age (years)	0.97	(0.93-1)	0.072
**Educational level**			
Up to high school degree	1.86	(0.73-4.77)	0.195
Bachelor’s degree	1.07	(0.37-3.1)	0.904
Completed postgraduation	1		
**Civil status**			
Single	2.04	(0.93-4.47)	0.074
Married/Stable union	1		
**Duration of membership plan**			
1-month	3.43	(1.53-7.68)	0.003
3-month to 6-month	1.47	(0.58-3.74)	0.419
Annual	1		
**Frequency during week one**	0.66	(0.5-0.87)	0.004
Diurnal preference HO (MEQ score)	0.99	(0.96-1.02)	0.571
Sleep quality (score)	1.11	(1.02-1.21)	0.012

Notes: HR = Hazard ratio; CI = Confidence interval; MEQ = Morningness-eveningness questionnaire.

## DISCUSSION

In the present study, we demonstrated for the first time that worse SQ is associated with higher risk of dropping out of PE programs during the first six weeks - the critical period for the formation of PE habits in the context of gyms^7^. Each PSQI point increase corresponded to an 11% increase in dropout risk, even adjusting for potential confounding variables.

Previous studies indicate that different aspects of sleep predicts PA execution on the next day^6^. Lower SQ the night before predicted lower leisure-time PA the next day in elderly people^14^. Holfeld and Ruthig (2014)^15^ demonstrated that worse baseline SQ predicted less total PA two years later in elderly people. Huang et al. (2021)^16^ in a cohort carried out in the United Kingdom, demonstrated that less healthy sleep predicted less total PA six years later, in a sample of 38,601 participants. It is possible that our data, even if limited to PA performed in leisure time in a formal context, partially portraits the challenge of people with poor SQ to engage in PE programs. This could partially help explain the SQ prediction of total PA found in longitudinal studies.

In another investigation, currently under review, we tested whether the chronotype could predict the PE drop out in three months and found that while the SQ did not predict it, the chronotype did. Apparently, people with poor SQ would have more difficulties during the first few weeks, while more evening-type people had more difficult during the first three months. People who reached the third month of exercise program improved their SQ significantly, which did not occur with dropouts before this period (unpublished data). Due to the fact that regular practice of PE improves SQ, possibly because of this SQ was not a predictor for a period longer than six weeks.

A possible explanation for our results could be a greater daytime sleepiness and less enthusiasm within the dropout group. The daytime dysfunction component of the PSQI differed significantly between groups, while other components taken individually did not (data not reported). Although we have not measured daytime sleepiness nor any psychological aspects, it is possible that during the follow-up the dropouts suffered greater fatigue, therefore being less willing to exercise. Daytime fatigue would constitute an additional barrier at the beginning of PE programs engagement^17^.

The results of the present study reinforce the importance of interdisciplinary approaches to favor the creation of PE habits. Recommendations to promote sleep health could help minimize the chances of early dropout related to poor SQ^18^. Recommendations in the context of gyms is an open and promising field, considering the high prevalence of sleep complaints in different cultures^19^ and the growing increase in the fitness market in many countries.

We believe that the assessment of the PA level reported by the volunteers may have been influenced by the practice already started in the gym since the questionnaires were filled out within one to three weeks after enrolling; although it was requested that the responses refer to the period prior to admission. On the other hand, as the gyms did not offer other services besides PE programs, the PE practice measure from the electronic turnstiles was reliable.

## CONCLUSION

The results of this study indicate that the perception of SQ predicts participation in supervised PE programs during the first six weeks of practice. Higher frequency of practice in the first week and the term of the plan contracted in gyms also predicted higher early withdrawal from PE programs.
